# Lipid Nanoparticle-Encapsulated TALEN-Encoding mRNA Inactivates Hepatitis B Virus Replication in Cultured Cells and Transgenic Mice

**DOI:** 10.3390/v17081090

**Published:** 2025-08-07

**Authors:** Tiffany Smith, Prashika Singh, Ridhwaanah Bhana, Dylan Kairuz, Kristie Bloom, Mohube Betty Maepa, Abdullah Ely, Patrick Arbuthnot

**Affiliations:** Wits/SAMRC Antiviral Gene Therapy Research Unit, Infectious Diseases and Oncology Research Institute (IDORI), School of Pathology, Faculty of Health Sciences, University of the Witwatersrand, Parktown 2193, South Africa; tiffany.smith@wits.ac.za (T.S.); prashika.singh.ps@gmail.com (P.S.); ridhwaanah.jacobs@gmail.com (R.B.); dylan.kairuz@students.wits.ac (D.K.); kristie.bloom@wits.ac.za (K.B.); betty.maepa@wits.ac.za (M.B.M.); abdullah.ely@wits.ac.za (A.E.)

**Keywords:** HBV, cccDNA, HBsAg, core, TALEN mRNA, LNP

## Abstract

Chronic infection with the hepatitis B virus (HBV) results in over 1 million deaths annually. Although currently licensed treatments, including pegylated interferon-α and nucleoside/nucleotide analogs, can inhibit viral replication, they rarely eradicate covalently closed circular DNA (cccDNA) reservoirs. Moreover, vaccination does not offer therapeutic benefit to already infected individuals or non-responders. Consequently, chronic infection is maintained by the persistence of cccDNA in infected hepatocytes. For this reason, novel therapeutic strategies that permanently inactivate cccDNA are a priority. Obligate heterodimeric transcription activator-like effector nucleases (TALENs) provide the precise gene-editing needed to disable cccDNA. To develop this strategy using a therapeutically relevant approach, TALEN-encoding mRNA targeting viral core and surface genes was synthesized using in vitro transcription with co-transcriptional capping. TALENs reduced hepatitis B surface antigen (HBsAg) by 80% in a liver-derived mammalian cell culture model of infection. In a stringent HBV transgenic murine model, a single dose of hepatotropic lipid nanoparticle-encapsulated TALEN mRNA lowered HBsAg by 63% and reduced viral particle equivalents by more than 99%, without evidence of toxicity. A surveyor assay demonstrated mean in vivo HBV DNA mutation rates of approximately 16% and 15% for Core and Surface TALENs, respectively. This study presents the first evidence of the therapeutic potential of TALEN-encoding mRNA to inactivate HBV replication permanently.

## 1. Introduction

Chronic infection with hepatitis B virus (HBV) is a growing global health concern with 296 million individuals affected worldwide [1,2]. It remains a leading cause of liver-related mortality, primarily a result of complicating cirrhosis and hepatocellular carcinoma (HCC) [3,4]. The virion carries a relaxed circular DNA (rcDNA) genome, which is transported to the hepatocyte nucleus after infection, then repaired to form covalently closed circular DNA (cccDNA) [5]. As a stable template for viral gene transcription, cccDNA serves as a reservoir that maintains persistent HBV replication [6]. In addition, HBV evades immune detection in chronic carriers. The viral genome is highly compact, encoding overlapping *precore*/*core* (C), *polymerase* (pol), *surface* (S), and *X* open-reading frames (ORFs) [7]. This arrangement limits HBV genome plasticity and is a feature that may be exploited in the development of sequence-specific candidate therapies.

Currently available antiviral therapies do not eliminate cccDNA, which results in the rebound of viral replication on treatment withdrawal [8,9]. As a result, patients generally require lifelong treatment to achieve durable viral replication and halt disease progression. In recent years, innovative antiviral therapies have been developed in an attempt to reduce cccDNA pools [10,11,12]. Bulevirtide, sold under the name Hepcludex, is a viral entry inhibitor that reduces viral loads in HBV infection models. Nucleic acid polymers (NAPs) are another group of promising therapies. These compounds selectively block the secretion of hepatitis B surface antigen (HBsAg), a key marker of viral replication. Nevertheless, these new drugs do not target cccDNA directly, and reactivation of viral replication may occur following treatment cessation.

The use of gene editing technologies is a promising approach to targeting HBV cccDNA [13,14,15]. Zinc finger nucleases (ZFNs) were among the earliest gene editing technologies developed. Two independent investigations demonstrated their ability to bind to target HBV DNA and modulate gene expression selectively [13,16]. Because of their design constraints, ZFNs are infrequently employed, especially in HBV targeting applications. Transcription activator-like effector nucleases (TALENs) are capable of precisely creating double-strand breaks (DSBs) at specific sites. TALENs comprise a DNA-binding domain, which is designed to bind to specific nucleotides in any given sequence of DNA, and a *Fok*I nuclease domain that is active as a dimer [17,18]. Although CRISPR/Cas9 is widely used for gene editing, pre-existing adaptive immunity to Cas9 derived from commensal microorganisms may attenuate editing efficiency [19,20]. TALENs’ direct action on DNA without a requirement for an RNA guide, DNA, and protospacer adjacent motif (PAM) [21] could also contribute. Additionally, CRISPR/Cas9 may be more prone to off-target effects and has constrained editing efficiency at heterochromatin regions [22]. Repeated cutting commonly induces error-prone DNA repair by non-homologous end joining (NHEJ), which leads to targeted permanent mutation of viral sequences. As a result of the compact HBV genome, TALEN-induced mutations may thus impact the function of multiple genes to disrupt viral replication significantly.

The successful targeting of HBV cccDNA by TALENs has been well-established [14,23,24]. Moreover, the approach using obligate heterodimeric TALENs has reduced off-target cleavage. Obligate heterodimeric TALENs comprise second-generation *Fok*I nuclease domains, which are not functional when homodimerization of TALEN monomers occurs [24]. These TALEN sequences were previously reported to disrupt HBV cccDNA with high specificity when expressed from plasmid templates. Building on this prior work, we designed TALEN-encoding mRNA to develop a more clinically relevant approach. mRNA offers a safer alternative to DNA because of the lack of genotoxicity and the short half-life that results in better dose control [25]. Moreover, mRNA is efficiently delivered to the cytoplasmic site of action using ionizable lipid nanoparticles (iLNPs), which also afford protection to the payload [26].

In this study, we designed TALEN-encoding mRNA to target the viral *core* and *surface* ORFs, referred to as Core and Surface TALENs, respectively. These gene editors also bind the overlapping *polymerase* ORF. We demonstrate for the first time that TALEN-encoded mRNA effectively reduces viral replication in several cell culture models, as well as in a stringent murine model of HBV replication.

## 2. Materials and Methods

### 2.1. Construction of mRNA Expression Plasmids

Plasmids were designed as templates for in vitro transcription (IVT) of mRNA encoding second-generation obligate heterodimeric TALENs, which targeted the *core* and *surface* genes of HBV. The TALE array sequences, located immediately downstream of the hemagglutinin (HA) epitope and a nuclear localization signal (NLS), were fused to second-generation *Fok*I nuclease domains that made up the complete TALENs. The nuclease domains had Q486E and I499A substitutions in the left monomer, and E490K and I538V substitutions in the right monomer [27]. These previously described TALEN sequences [25] were excised from pVAX plasmids and inserted into a standard mRNA expression vector that contained a T7 promoter sequence, 5′ untranslated region (UTR), 3′ UTR, and polyA coding sequence. A unique restriction site, positioned downstream of the poly (A) sequence, was used to linearize the plasmid. The cloned portions of all TALEN plasmid constructs were verified using DNA sequencing. Additionally, mCherry and firefly luciferase (FLuc) sequences were individually inserted into the mRNA expression vectors and served as markers of expression and mock controls.

### 2.2. mRNA Production

mRNA was produced as previously described [28]. In brief, linearized templates were transcribed in vitro using T7 RNA polymerase (High concentration; New England Biolabs, Ipswich, MA, USA). N-methylpseudouridine (m1Ψ) (TriLink Biotechnologies, San Diego, CA, USA) and CleanCap^®^ AG (TriLink Biotechnologies, San Diego, CA, USA) were incorporated co-transcriptionally. The resulting mRNAs were treated with DNase I (New England Biolabs, Ipswich, MA, USA) and recovered using lithium chloride precipitation. To remove double-stranded RNA, cellulose-based purification was carried out as previously described [29]. Spectrophotometry was used to determine RNA concentration and purity, and a 1% denaturing formaldehyde gel was employed to evaluate RNA integrity.

### 2.3. Cell Culture

Huh7 cells were cultured in low-glucose (1 g/L) Gibco™ Dulbecco’s Modified Eagle’s Medium (DMEM) (Thermo Fisher Scientific, Waltham, MA, USA), and HepG2.2.15 cells were cultured in high-glucose DMEM. Growth media contained penicillin (100,000 U/mL), streptomycin (100 µg/mL), and 10% heat-inactivated fetal bovine serum (FBS) (Sigma–Aldrich, St. Louis, MO, USA). HepG2-hNTCP-C4 cells from the National Institute of Infectious Diseases (Tokyo, Japan) [30] were cultured in DMEM/F12 with GlutaMAX™ (Thermo Fisher Scientific, Waltham, MA, USA) and supplemented with 10% FBS, penicillin (200,000 U/mL), streptomycin (200 µg/mL), 10 mM HEPES, 50 mM hydrocortisone, insulin (5 µg/mL), and G418 (400 µg/mL). Cells were incubated at 37 °C with 5% CO_2_ in a humidified incubator.

### 2.4. Immunocytochemistry Detection of TALEN Transcripts

One day before transfection, Huh7 cells were seeded in a 48-well plate at a cell density of 70% per well. Lipofectamine™ MessengerMAX™ (Invitrogen, Carlsbad, CA, USA) was used to transfect cells with TALEN or mCherry transcripts (mock control). Six hours after transfection, TALEN expression was measured by detecting the HA epitope using an anti-HA primary antibody (Sigma–Aldrich, St. Louis, MO, USA) and an Alexa Fluor 488-conjugated secondary antibody (Thermo Fisher Scientific, Waltham, MA, USA). Fluorescence images were captured with the EVOS^®^ XL Cell Imaging System (Invitrogen, Carlsbad, CA, USA).

### 2.5. Measuring HBsAg Secretion from Liver-Derived Cells Using ELISA

Two liver-derived cell lines, Huh7 and HepG2-hNTCP-C4, were employed to assess TALEN effectiveness in vitro. Huh7 cells were transfected with pCH-9/3091 [31] (an HBV replication-competent plasmid). This construct harbors a greater-than-genome-length HBV sequence based on genotype D, which facilitates the transcription of pgRNA in transfected cells. Subsequent reverse transcription of pgRNA initiates the formation of cccDNA. Huh7 cells were also transduced with adeno-associated virus serotype 2-containing a greater-than-genome length (1.28×) replication-competent HBV genome sequence from subgenotype D3 (AAV2-D3) [32]. For studies using Huh7 and pCH-9/3091, cells were seeded in a 48-well plate at a density of 50% one day before transfection. Using Lipofectamine™ 3000 (Invitrogen, Carlsbad, CA, USA), 50 ng of pCH-9/3091 was delivered to the cells and incubated for one hour. Following this, cells were transfected with 225 ng of each Left and Right TALEN mRNA. In separate wells, as a mock control, cells were transfected with 450 ng of mCherry mRNA. Transfection success was verified by visualizing mCherry expression and quantifying hepatitis B surface antigen (HBsAg) secretion 24 and 48 h after transfection. Fluorescence microscopy was used to identify mCherry expression, while ELISA was used to measure HBsAg secretion using the Monolisa™ HBsAg ULTRA kit (Bio-Rad, Hercules, CA, USA).

#### 2.5.1. Huh7 and AAV2-D3

Recombinant AAV2 vectors, carrying HBV DNA from subgenotype D3, were generated as previously described [32]. Huh7 cells were seeded at a cell density of 50% in a 24-well plate the day before infection. The cells were subsequently transduced with AAV2-D3 at a multiplicity of infection (MOI) of 1000 and incubated for 48 h. Cells were co-transfected with 500 ng of Left and Right TALEN transcripts. In separate wells, cells were transfected with 1 µg of mCherry mRNA. mCherry expression was verified, and HBsAg secretion was assessed 72 and 96 h after infection.

#### 2.5.2. HepG2-hNTCP-C4

HBV was prepared for infection of HepG2-hNTCP-C4 cells as previously described [33]. Briefly, genotype D HBV was obtained from HepG2.2.15 cells. HepG2-hNTCP-C4 cells were seeded at a cell density of 65% in 24-well collagen-coated plates (Thermo Fisher Scientific, Waltham, MA, USA). After 24 h, the cells were treated with DMEM/F12 containing 2% dimethyl sulfoxide (DMSO; Sigma–Aldrich, St. Louis, MO, USA). After 48 h, the cells were infected with HBV at an MOI of 10 in a solution containing 2% DMSO and 4% polyethylene glycol (PEG; Sigma–Aldrich, St. Louis, MO, USA) in DMEM/F12. Following 8 h of infection, cells were co-transfected with 500 ng of each Left and Right TALEN transcripts. In separate wells, cells were transfected with 1 µg of mCherry mRNA. mCherry expression and HBsAg secretion were assessed 24 h after infection.

### 2.6. In Vitro Cell Viability Assay

Twenty-four hours before transfection, Huh7 and HepG2-hNTCP-C4 cells were seeded in separate 96-well plates at a cell density of 50%. Cells were then co-transfected with 50 ng of each Left and Right TALEN transcripts. As a mock control, cells were transfected with 100 ng of mCherry mRNA. Positive and negative control cells were treated with 50% DMSO or untreated, respectively. Cell viability was measured 24 h after transfection by adding 20 µL of 5 mg/mL MTT (Invitrogen, Carlsbad, CA, USA) to the wells and incubating at 37 °C for 1 h. Culture medium was removed, and 200 µL of DMSO was added and incubated for no more than 5 min. The absorbance was measured at 570 nm and 655 nm using an iMARK™ Microplate reader (Bio-Rad, Hercules, CA, USA).

### 2.7. Lipid Nanoparticle (LNP) Formulations

#### 2.7.1. LNP-Encapsulation

LNPs were formulated by mixing an ethanolic lipid solution with mRNA in an acidic aqueous buffer, as previously outlined [34]. Briefly, SM-102, DSPC, cholesterol, and DMG-PEG 2000 were dissolved in ethanol at a final molar ratio of 50:10:38.5:1.5 [34,35]. FLuc-, mCherry-, and TALEN-encoding mRNAs were diluted in sodium acetate buffer (6.25 mM, pH 5). The lipid solution was mixed with the mRNA solution at a flow rate of 1:3 (ethanol/citrate, *v*/*v*) using a NanoAssemblr^®^ Ignite^TM^ system (Precision NanoSystems, Vancouver, Canada), to form LNPs with a nitrogen-to-phosphorus (N/P) ratio of 1:6. The LNP formulation was diluted 40 times with 1× PBS (pH 7.4) and concentrated using Amicon^®^ Ultra-15 centrifugal filters (100 kDa MWCO; Sigma–Aldrich, St. Louis, MO, USA). Centrifugation was carried out using a swing-bucket rotor at 1000× *g* for 10–15 min. The LNPs were sterilized by filtration through Acrodisc^®^ Supor^®^ membranes (Pall Corporation, Port Washington, NY, USA).

#### 2.7.2. LNP Size and Surface Charge Determination

For size and zeta potential measurements, LNPs were diluted 200-fold in nuclease-free water. Size measurements were conducted using a clear rectangular cuvette in the ZetaSizer ZS Xplorer (Malvern Panalytical Ltd., Malvern, UK) under default settings. Zeta potential was measured using a cuvette with gold-plated electrodes and a 1 mL syringe, also in the ZetaSizer under default conditions. Particle sizes ranged from 66.57 to 88.3 nm, with polydispersity indices below 0.2, and zeta potential values between −1.665 and 8.812 mV, indicating a neutral charge (Table S1).

#### 2.7.3. RNA Quantification and Encapsulation Efficiency

The Quant-iT RiboGreen RNA Kit (Thermo Fisher Scientific, Waltham, MA, USA) was used to determine encapsulation efficiency according to the PNI RiboGreen^®^ Assay Protocol (Precision NanoSystems, Vancouver, Canada). Fluorescence measurements were carried out using the GloMax^®^ Explorer (Promega, Madison, WI, USA) at excitation wavelengths of 500–550 nm. Encapsulation efficiency (%) was measured by comparing the fluorescence intensity of unencapsulated RNA in the absence of detergents (F_i_) with the total RNA fluorescence intensity (F_t_) after LNP lysis with Triton-X100 (Sigma–Aldrich, St. Louis, MO, USA). Encapsulation Efficiency (%) was calculated as (F_t_ − F_i_/F_t_ × 100).

### 2.8. Animal Studies

Friend leukemia virus B (FVB) HBV transgenic mice were used for this study [36], with all animal procedures approved by the University of the Witwatersrand Animal Research Ethics Committee. Mice received a 2 mg/kg dose of LNP-encapsulated Core TALENs, Surface TALENs, or FLuc/mCherry by intravenous injection into the lateral tail vein. Mice were peritoneally injected with 150 mg/kg D-luciferin (Perkin Elmer Inc., Waltham, MA, USA) at 6- and 24-h time points after LNP administration. Bioluminescence imaging was performed with the IVIS Kinetic In Vivo Optical Imaging System (Perkin Elmer Inc., Waltham, MA, USA). Blood samples were taken on days 1, 8, and 15 for serum analysis. ELISA was performed on serum from days 1, 8, and 15. Alanine aminotransferase (ALT) and aspartate aminotransferase (AST) activities were also measured in serum samples collected at day 15. These assays were carried out at the National Health Laboratory Service (NHLS) at Charlotte Maxeke Academic Hospital, Johannesburg, using the Advia 1800 Chemistry System (Siemens Healthineers, Erlangen, Germany). Viral particle equivalents (VPEs) were measured separately on day 15. Mice were euthanized on day 15 via CO_2_ exposure, and total RNA was extracted from 100 mg of liver tissue.

### 2.9. Quantification of Circulating VPEs, HBV Core, and Surface Genes

To quantify VPEs, real-time qPCR was performed on serum samples collected on day 15 after a 1:8 dilution in saline. Total DNA was extracted from experimental and control mouse sera using the QIAamp DNA Mini Kit (Qiagen, Hilden, Germany) and analyzed using the CFX96™ Real-Time instrument (Bio-Rad, Hercules, CA, USA). Ten-fold serial dilutions of pCH-9/3091 were used as standards for quantitation. The FastStart Essential DNA Green Master Kit (Roche Applied Science, Mannheim, Germany) was used to amplify DNA samples, with the following primer sets: HBV Surface Forward: 5′-TGC AAC TGT ATT CCC ATC-3′ and HBV Surface Reverse: 5′-CTG AAA GCC AAA CAG TGG-3′. To determine intrahepatic HBV gene expression, viral RNA was measured using Reverse Transcription qPCR. Total cellular RNA was isolated from liver homogenates using TRIzol^®^ Reagent (Thermo Fisher Scientific, Waltham, MA, USA). Reverse transcription and quantification were performed in a single reaction using the Luna^®^ Universal One-Step RT-qPCR Kit (New England Biolabs, Ipswich, MA, USA). Viral surface and pregenomic RNA (pgRNA) were amplified with HBV Surface and basal core promoter (Forward: 5′-ACC ACC AAA TGC CCC TAT-3′ and Reverse: 5′-TTC TGC GAG GCG GCG A-3′), respectively. To standardize viral RNA levels, murine *GAPDH* mRNA was utilized as a reference, and amplification was carried out with the following primers: mGAPDH Forward: 5′-TTC ACC ACC ATG GAG AAG GC-3′ and mGAPDH Reverse: 5′-GGC ATG GAC TGT GGT CAT GA-3′.

### 2.10. Assessing In Vivo Targeted Cleavage via Anti-HBV TALENs Using the Surveyor Nuclease Assay

To evaluate targeted cleavage in murine samples, a total of 25 mg of tissue was extracted from liver homogenates. Total DNA was extracted using the QIAamp DNA Mini Kit following the tissue protocol. Standard PCR conditions were used to amplify sequences comprising 520 bp of the target sites for the Core and Surface TALENs. The sequences from FLuc/mCherry-treated mice were also amplified with either core or surface primer sets. The following primer sets were used: Core forward 5′-GAA CTA ATG ACT CTA GCT ACC T-3′; Core reverse 5′-CCT ACA AAC TGT TCA CAT TT-3′; Surface forward 5′-CCT AGG ACC CCT TTC TCG TGT-3′, and Surface reverse 5′-ACT GAG CCA GGA GAA ACG GG-3′. PCR products were resolved on a 1% agarose gel and purified using the QIAquick^®^ Gel Extraction Kit (QIAGEN, Hilden, Germany). Three hundred and fifty nanograms of PCR products were subjected to heteroduplex formation under conditions previously described [37]. CelI was extracted from stalks of celery (*Apium graveolens*) through salting and dialysis methods previously described [38]. Two microliters of CelI extract were used per sample. Cleaved products were resolved using agarose gel electrophoresis, and ImageJ software (version 1.54g) was used to measure targeted disruption as previously described [39]. As a positive control for the Surveyor assay, heteroduplexes were formed after PCR amplification of wild-type and mutant HBx sequences derived from previously described psiCHECK-HBx constructs [40]. The following primer sets were used to amplify 514 bp fragments of HBx: HBx forward 5′-CGA TAA GCT TGA TAT CGA ATT CCA T-3′ and HBx reverse 5′-CAG TGG GAC ATG TAC AAG AGA ATG A-3′. HBx amplicons were mixed at equimolar amounts and denatured, then annealed to form heteroduplexes.

### 2.11. Assessing TALEN-Induced Innate Immune Stimulation in Mice

Day 15 serum samples were collected from mice to assess inflammatory responses. Mice were divided into five groups, each receiving a tail vein injection of either 5 mg/kg polyinosinic:polycytidylic acid (Poly I:C) (positive control), 150 µL saline (negative control), 2 mg/kg LNP-encapsulated Core TALENs, Surface TALENs, or FLuc/mCherry (mock group). Cytokine levels were evaluated using the Cytometric Bead Array (CBA) Mouse Kit (BD BioSciences, San Jose, CA, USA), measuring four cytokines: tumor necrosis factor-α (TNF-α), Monocyte Chemoattractant Protein-1 (MCP-1), interleukin-6 (IL-6), and IL-10. The assay was performed in a 96-well V-bottom plate (Thermo Fisher Scientific, Waltham, MA, USA) using the BD Accuri™ C6 flow cytometer (BD Biosciences, San Jose, CA, USA). Data were quantified using BD^®^ CBA Analysis Software (v3.0).

### 2.12. Statistical Analysis

All experiments were conducted in biological triplicate. For statistical analysis, the mean and standard error of the mean (SEM) were computed and plotted for each dataset. Relative light units (RLUs) of mice receiving FLuc mRNA, determined following bioluminescence, and ALT and AST values were plotted for individual mice. A two-tailed Student’s *t*-test was used to establish statistical significance, which was performed using GraphPad Prism 8.0 (GraphPad Software Inc., La Jolla, CA, USA). A *p*-value of <0.05 was considered statistically significant.

## 3. Results

### 3.1. Producing Anti-HBV TALEN-Encoding mRNA

**Figure 1:**
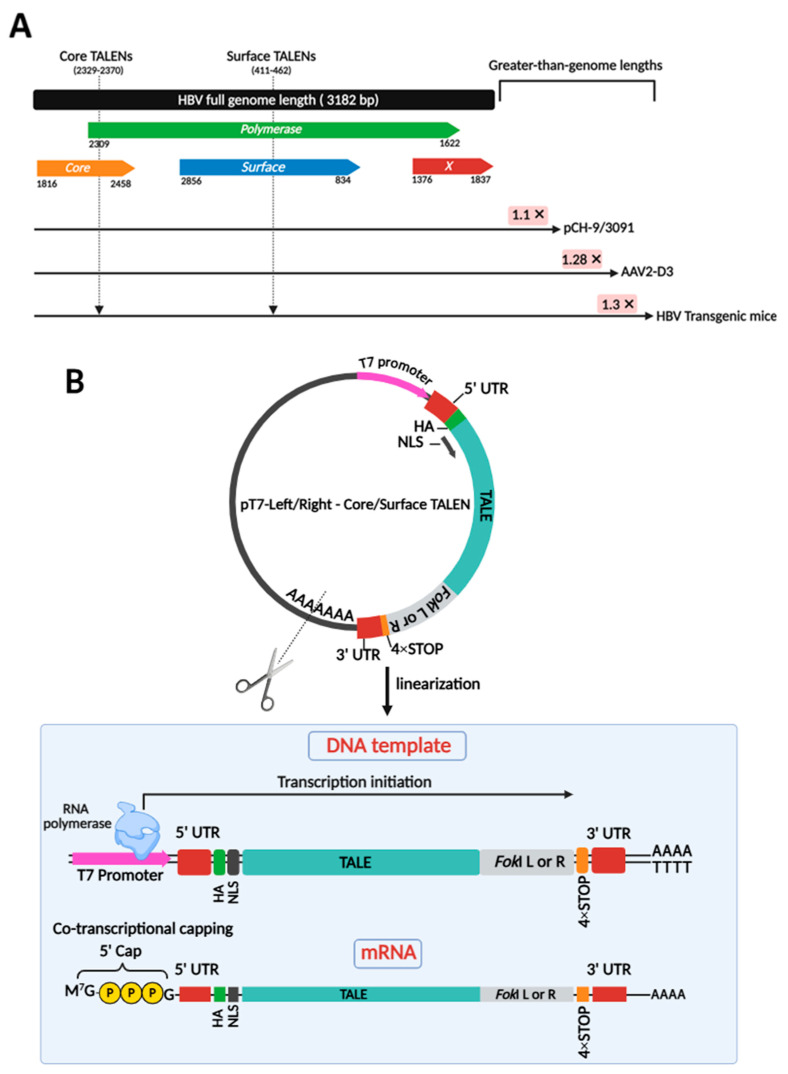
Target sites on the HBV genome for obligate heterodimeric cleavage and generation of TALEN-encoding mRNA targeting HBV. (**A**) The linearized genomic organization of HBV comprises four open reading frames (ORFs) designated as *Core*, *Polymerase*, *Surface*, and *X*, spanning approximately 3.2 kb. Greater-than-genome lengths are depicted for HBV sequences in pCH-9/3091, AAV2-D3, and HBV transgenic mice. Two obligate heterodimeric TALENs were generated to target both the *core* and *surface* ORFs of HBV DNA. These sites are indicated by arrows. These TALENs also target the overlapping *polymerase* ORF. (**B**) Plasmid templates encoding TALEN sequences were linearized to serve as transcription templates for IVT. Co-transcriptional capping resulted in mature mRNA strands, ready for translation.

Two obligate heterodimeric TALENs were employed to target *core* and *surface* ORFs of HBV DNA in various models of infection, including plasmid-derived pCH-9/3091, AAV-derived AAV2-D3, and integrated HBV in transgenic mice (Figure 1A). Targeting a range of models is crucial to demonstrate that TALENs are effective across diverse contexts of HBV persistence, whether episomal or chromosomally integrated. To produce clinically relevant anti-HBV gene editors, TALEN-encoding mRNAs were successfully generated by cloning previously designed TALEN-encoding sequences [24] into a pT7-based expression vector optimized for in vitro transcription (IVT) (Figure 1B). This vector included a 4× STOP codon sequence at the 3′ end of the ORF and upstream of the 3′ UTR. This was included to prevent read-through errors and enhance translation. Plasmids were linearized (Figure S1) and co-transcriptionally capped with modified cap-1 for enhanced stability and translation efficiency (Figure 1B). Obligate heterodimeric cleavage, owing to second-generation *Fok*I nuclease domains, enhances the safety profile of these TALENs. The TALENs targeting the HBV *core* and *surface* ORFs were initially tested for efficacy in cultured cells.

### 3.2. TALEN mRNA-Mediated Inhibition of HBsAg Secretion in HBV Cell Culture Models

**Figure 2:**
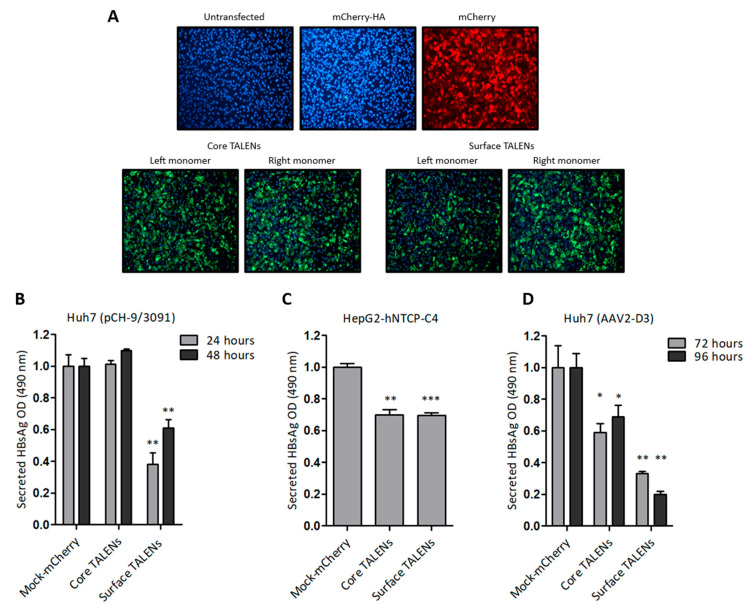
TALEN efficacy in cultured liver-derived cells: (**A**) Immunofluorescence detection of HA tag fused to TALENs in Huh7 cells following transfection with left or right TALEN transcripts. mCherry mRNA and untransfected cells served as negative controls. Cell nuclei were counterstained with DAPI, and representative images were captured at 20× magnification. (**B**) ELISA quantification of HBsAg secretion in culture supernatants of Huh7 cells after co-transfection with pCH-9/3091 and TALEN transcripts. HBsAg secretion was measured 24 and 48 h after transfection. (**C**) HepG2-hNTCP-C4 cells infected with HBV and treated with TALENs were assessed for HBsAg secretion 24 h after transfection. (**D**) HBsAg secretion from AAV-D3-transduced Huh7 cells following TALEN treatment. Measurements were made 72 and 96 h after transfection. Data are presented as mean values ± SEM. Statistical significance was determined relative to mock controls (*: *p* < 0.05, **: *p* < 0.01, ***: *p* < 0.001).

Huh7 cells were initially transfected with TALEN-encoding mRNA to assess protein expression. Immunofluorescence detection revealed nuclear localization of the HA epitope six hours after transfection (Figure 2A), which confirmed the successful translation of the TALEN transcripts. TALEN efficacy was next assessed in several cell culture models of HBV replication. Huh7 cells were transfected with pCH-9/3091, an HBV replication-competent construct. HBsAg secretion was measured following TALEN-encoding mRNA administration. Culture supernatants demonstrated a significant decrease in secreted HBsAg, with a 62% reduction observed at 24 h after transfection of cells with mRNA encoding the Surface TALENs (Figure 2B). As anticipated, HBsAg levels increased at the 48-h time point, which is attributed to continual replication from the pCH-9/3091 plasmid [31]. Importantly, the observed reduction in secreted HBsAg confirmed the action of the Surface TALENs on the targeted *surface* ORF, a critical region for HBV replication [41]. Core TALENs did not affect HBsAg secretion as expected.

To investigate the efficacy of TALENs in a physiologically relevant cell culture model, HepG2-hNTCP-C4 cells stably expressing the sodium taurocholate co-transporting polypeptide (NTCP) receptor [30] were infected with HBV, then transfected with mRNA encoding the TALENs. Surface TALENs resulted in a modest reduction of secreted HBsAg, and the efficacy of Core TALENs was unexpectedly similar to that of *surface*-targeted TALENs (Figure 2C). This indicates that Core TALENs may impede viral protein expression indirectly. Additional validation was carried out in Huh7 cells transduced with the HBV replication-competent adeno-associated viral vector, AAV2-D3. In this context, the TALEN function was monitored up to 4 days following transduction. Surface TALENs demonstrated a superior reduction in HBsAg levels by 70% at 72 h, and a sustained decline to 80% at 96 h was then observed (Figure 2D). Although Core TALEN-encoding mRNAs also effected significant decreases in HBsAg by 40% at 72 h, secreted HBsAg increased by 96 h.

Viability assays were used for all cell culture studies to exclude confounding cytotoxicity. No significant cytotoxic effects were observed with any of the analyses, supporting the hypothesis that TALEN-mediated inhibition of HBsAg secretion occurs without causing widespread cellular damage (Figure S2).

### 3.3. LNP-Encapsulated TALEN-Mediated Inhibition of HBV Replication in Transgenic Mice

**Figure 3:**
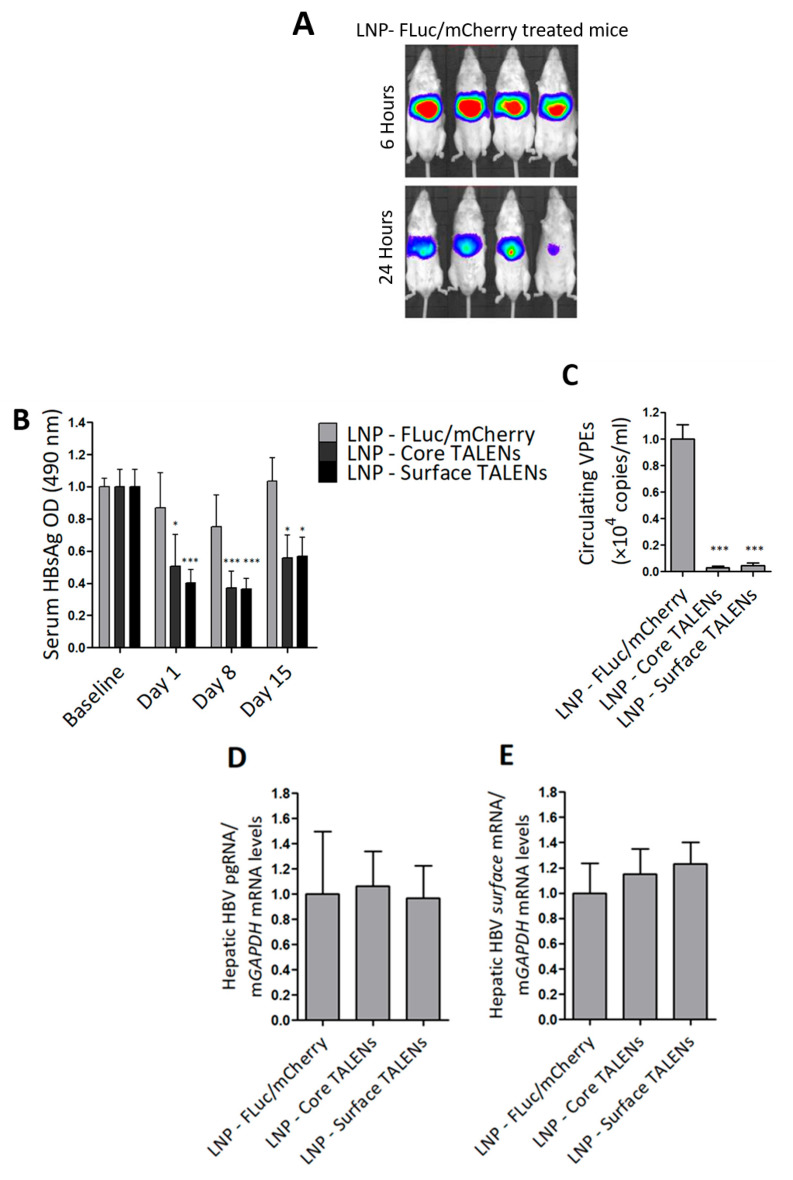
TALENs suppress HBV replication in transgenic mice. HBV transgenic mice received a single intravenous dose of LNP-encapsulated TALEN mRNA or LNPs containing FLuc/mCherry-encoding mRNA as a control: (**A**) Bioluminescence imaging at 6 h and 24 h after mice received FLuc/mCherry mRNA. (**B**) HBsAg secretion in serum was quantified on days 1, 8, and 15 after injection of the LNPs. Data were normalized to baseline optical densities (ODs). (**C**) Circulating viral particle equivalents (VPEs) were measured using qPCR analysis carried out on total DNA extracted from serum samples. (**D**) Intrahepatic pregenomic RNA (pgRNA) and (**E**) surface mRNA levels were quantified using RT-qPCR and normalized to *mGAPDH*. Data are presented as mean values ± SEM, with statistical significance determined by comparing to mock controls (*: *p* < 0.05, ***: *p* < 0.001). Sample sizes: LNP-FLuc/mCherry (n = 4), LNP-Core TALEN (n = 8), LNP-Surface TALEN (n = 8).

HBV transgenic mice were used to determine the efficacy of TALEN-encoding mRNAs in an in vivo setting. The mice contain a greater-than-genome-length HBV sequence genotype D subtype ayw stably integrated into their genomes, which enables the constitutive replication of the virus in mouse hepatocytes [36]. The study was evaluated over a 15-day period. LNP-encapsulated TALENs or LNPs containing FLuc/mCherry were administered to mice by tail vein injection. Bioluminescence imaging was used to confirm hepatic delivery in mice receiving the FLuc transcript. As predicted, bioluminescence imaging was detected exclusively in the liver within 6 h of injection, which persisted at 24 h (Figure 3A and Figure S3). These findings validated successful hepatic delivery and stability of the mRNA following delivery with LNPs.

Next, markers of replication were assessed in serum samples collected at multiple time points. Because transgenic mice already express moderate to high levels of HBsAg, baseline serum was measured to determine the anti-HBV effects from TALENs accurately. Both Surface and Core TALENs significantly reduced HBsAg secretion (Figure 3B). In contrast, mock-treated mice exhibited no significant reduction in HBsAg levels, with only a transient decrease observed on day 8. While reduced HBsAg secretion was observed in mice treated with the Core TALENs, this is in line with the results from cultured cells (Figure 2C,D). Notably, we have previously reported inhibition of HBsAg in a non-transgenic murine model of HBV replication by Core TALENs [14,24].

To further evaluate the impact of TALEN-encoding mRNA on hepatitis B virion production, circulating VPEs were quantified from serum collected on day 15. Both Core and Surface TALENs achieved >99% suppression of circulating VPEs compared to mock-treated mice (Figure 3C). This reduction in VPEs is consistent with the mechanisms by which each TALEN operates. Core TALENs inhibit capsid assembly to prevent encapsidation of pregenomic RNA (pgRNA) and the subsequent formation of infectious virions, whereas Surface TALENs affect the translation of surface antigens required for virion secretion. Moreover, this remarkable decrease further validates the key capability of TALENs to induce permanent mutations.

Viral gene expression was assessed through quantitation of intrahepatic HBV mRNA using RT-qPCR. Compared to levels observed in control animals, neither Core nor Surface TALENs significantly affected HBV mRNA expression (Figure 3D,E). This is expected as these TALENs were not designed to bind to transcriptional regulatory elements. While the observed suppression of replication is likely to be because of targeted mutagenesis rather than transcriptional suppression, it should be noted that these findings are derived from transgenic chromosomal target sequences and may not fully represent transcriptional mechanisms in rcDNA or cccDNA. The data also highlight that inhibition of HBsAg secretion by Core TALENs must occur through the inhibition of protein expression.

### 3.4. Targeted Mutagenesis by TALENs In Vivo

**Figure 4:**
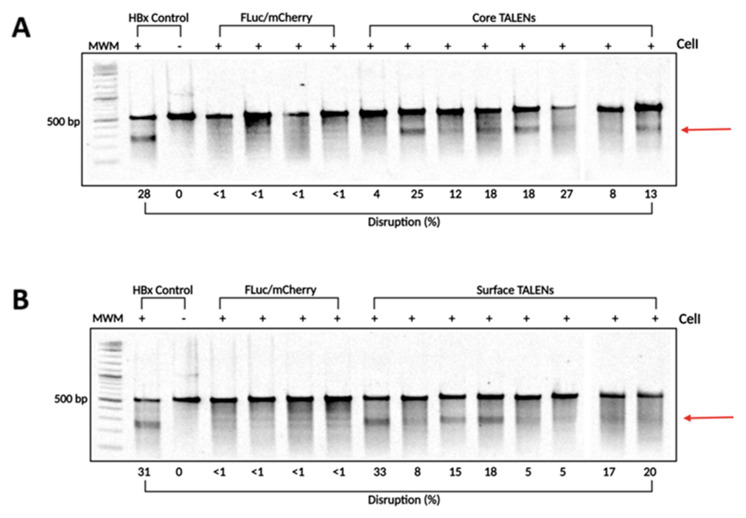
TALEN-mediated targeted disruption of HBV-encoding DNA in transgenic murine liver samples. To determine the levels of targeted mutagenesis mediated by the TALENs in vivo, total DNA was extracted from transgenic murine livers. (**A**) Core and (**B**) Surface ORF target sites were amplified using PCR. The amplicons were subjected to CelI cleavage. As a positive control, HBx wild-type and mutant sequences were mixed in a 1:1 ratio and subjected to CelI cleavage. Arrows depict predicted on-target cleavage. MWM: molecular weight marker.

To confirm that TALENs caused targeted disruption within the HBV genome, DNA was extracted from liver samples collected on day 15 post-injection. The target sites of the TALENs were amplified using PCR to assess cleavage at intended sites. The resulting amplicons were analyzed using the surveyor nuclease assay. Successful cleavage of heteroduplexes indicated targeted mutagenesis at intended sites (Figure 4). Interestingly, Core and Surface TALENs demonstrated nearly identical cleavage disruption, with Core TALENs exhibiting targeted disruption of (15.62 ± 2.8%) (Figure 4A) and Surface TALENs (15.13 ± 3.3%) (Figure 4B). This further corroborates TALEN efficacy, evidenced by the nearly 100% reduction in VPE levels. The sequences amplified from FLuc/mCherry mice did not result in detectable cleavage, as expected.

### 3.5. LNP-Encapsulated TALENs Demonstrate Minimal Inflammatory Response and Absence of Liver Toxicity

**Figure 5:**
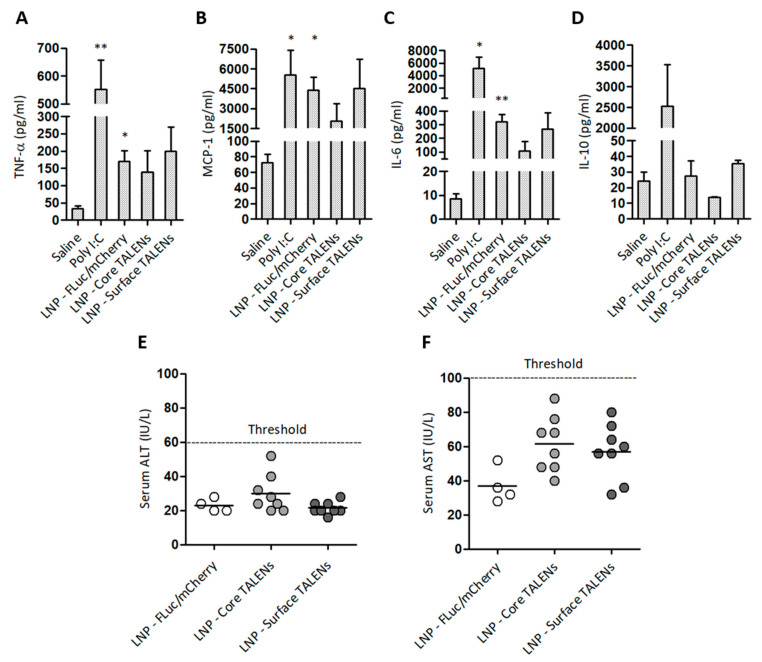
Safety profile of LNP-encapsulated TALEN-encoding mRNAs in mice: (**A**–**D**) Serum cytokine concentrations were measured 6 h after injection. Poly I:C and saline served as positive and negative controls, respectively. Data are presented as mean values ± SEM, with statistical significance compared to negative controls (*: *p* < 0.05, **: *p* < 0.01). Sample sizes: (n = 3) per group. (**E**) Serum ALT and (**F**) AST levels were quantified on day 15 to assess hepatotoxicity. Individual data points are shown, with horizontal bars indicating mean values.

The safety of LNP-encapsulated TALENs was assessed by measuring serum inflammatory markers and enzymes associated with liver injury in treated mice (Figure 5). Pro-inflammatory cytokine levels were measured 6 h after the administration of LNP-encapsulated TALEN or reporter mRNA. Injections with naked Poly I:C or saline were also included as controls (Figure 5A–D). TALENs demonstrated moderate increases in MCP-1 and IL-6 compared to saline controls. The increase in MCP-1 was anticipated given its role as an early marker of inflammation [42]. Unexpectedly, LNPs containing FLuc/mCherry resulted in significant increases in TNF-α, MCP-1, and IL-6. This could be attributed to an immunogenic response to the FLuc/mCherry transcripts themselves because LNP-encapsulated TALENs did not induce significant increases in cytokine concentrations. Poly I:C, the positive control, elicited increases that were similar to those of the LNP-FLuc/mCherry formulations. As expected, poly I:C also elevated serum IL-10 concentrations. Neither TALEN- nor FLuc/mCherry-encapsulated formulations led to concerning increases in ALT and AST activities (Figure 5E,F). These findings demonstrate that LNP-encapsulated TALENs have a good safety profile, with minimal inflammatory reactions and no significant hepatotoxicity.

## 4. Discussion

TALENs are capable of efficiently attenuating HBV replication in vivo, which supports their development for therapeutic application against the virus [14,24]. In a previous study, we used hydrodynamic injection to deliver DNA expression cassettes encoding the obligate heterodimeric Core and Surface TALENs. The gene editors achieved robust inhibition of HBV replication and improved targeted specificity. In the current study, we used LNP-formulated mRNA to encode similar TALEN sequences and achieved excellent inhibition of HBV replication in vivo.

Although TALENs are effective against HBV, most studies have reported on the use of naked plasmid DNA to express TALENs. This approach is not favored for clinical application because safe and efficient delivery to sufficiently large numbers of hepatocyte nuclei is difficult to achieve. Importantly, the previously used hydrodynamic injection mode of DNA delivery causes cytotoxicity and is unsuited to clinical translation. Progress with LNP-based delivery of plasmid DNA was recently described [43]; however, further development will be necessary before widespread application becomes a reality. Other drawbacks of using plasmid DNA are that the dose of therapeutic transgenes is difficult to regulate, plasmid DNA is prone to potentially genotoxic insertional mutagenesis, and inflammation-induced clearance. Recombinant viruses are efficient; however, they are also not without disadvantages. These include immunogenicity associated with pre-existing antibodies to viral capsids and restrictions to transgene cargo capacity [44,45]. In the case of therapeutic gene editing, the use of mRNA to encode designer nucleases is preferred. The shorter half-life of mRNA compared to DNA limits unwanted off-target effects that may result from long-lasting expression of gene editors. Previously, we carried out detailed next-generation sequencing analysis of the mutagenic effects of the Core and Surface TALENs, which were expressed from the DNA templates after hydrodynamic injection into mice [24]. Targeted mutation was verified, and minimal non-specific effects were observed. Given that the previous study employed TALEN-encoding DNA to evaluate antiviral efficacy in vivo, the use of the shorter-acting mRNA to encode the same TALENs is likely to be more specific. Absence of apparent toxicity also supports this assertion.

Core and Surface TALENs target different sites of the HBV genome. Surface TALENs target the *surface* ORF and, as anticipated, lead to significant reductions in HBsAg concentrations in cultured cells and in vivo. Despite targeting a different region, the Core TALEN also achieved reductions in HBsAg levels that were similar to *surface*-targeting TALENs. Reduction in HBsAg by Core TALENs has also been reported by others [14]. This suggests an indirect hindrance to the synthesis of the antigen. Because Core TALENs did not affect intrahepatic HBV mRNA levels, it is unlikely that transcriptional repression is responsible for reductions in HBsAg concentrations. Instead, Core TALENs’ effects on the *core* ORF are expected to induce mutations that interfere with virion assembly, and subsequent reduction in production of HBsAg-containing virions. Because ORFs of the HBV genome overlap with each other, Core and Surface TALENs also target *polymerase*. Polymerase is responsible for reverse transcription of pgRNA, and mutation of the essential enzyme should significantly hinder viral replication with concomitant reduction in HBsAg secretion. Investigating the effects of *core* knock-out clones on HBsAg production may reveal an indirect *core* dependency. Furthermore, both Core and Surface TALENs demonstrated targeted mutagenesis in HBV DNA isolated from transgenic mice. This supports the conclusion that TALEN efficacy arises from direct DNA cleavage and mutation according to the intended design. Although decreases in markers of HBV replication in transgenic mice, particularly circulating viral particle equivalents, were greater than the targeted mutation detected using the Surveyor assay, this may reflect limitations of the sensitivity of our assay. Possible explanations include partial CEL I cleavage and the presence of HBV DNA mutations that were undetected by the enzyme. These factors would cause an underestimation of mutation frequencies.

An ongoing challenge for the preclinical evaluation of new HBV curative therapies emanates from the dearth of suitable animal models that accurately mimic HBV infection and cccDNA formation. Chimpanzees are a well-characterized model of HBV infection [46]. They are capable of mimicking pathogenesis and disease progression observed in humans. However, ethical concerns have largely led to the worldwide prohibition of their use in biomedical research. Exorbitant maintenance costs also prevent their use for evaluating new anti-HBV therapeutics. Murine models with humanized livers have been developed to overcome some of these obstacles. Importantly, these models support ongoing HBV replication, which cannot be studied in transgenic or AAV-HBV-treated mice. However, mice with humanized livers are costly and present challenges, which include kidney disorders, low breeding efficiency, fatal bleeding, and immunodeficiency [47]. Recent advances with human liver organoids offer a promising alternative for modeling HBV infection [48,49]. Derived from healthy donor liver tissue, these organoids can be infected with HBV to produce cccDNA and provide a more physiologically relevant system for studying HBV dynamics. Nevertheless, the FVB-HBV transgenic murine model used here, though unable to produce cccDNA, remains valuable for studying the effects of candidate therapies on HBV replication [50]. Variability of serum HBsAg secretion is, however, a common limitation in this model and may explain the unexpected decreases in HBsAg that were observed in mock-treated mice. Nevertheless, the finding that both Core and Surface TALENs reduced circulating VPEs to barely detectable levels is a noteworthy indicator of therapeutic potential.

LNP-delivered TALEN mRNA displayed a safe toxicological profile, with limited activation of pro-inflammatory cytokines. Although LNP-encapsulated FLuc/mCherry elicited an innate immune response (Figure 4), this was not unexpected because recent research shows that a high dose of FLuc per se can induce robust innate immunity [51]. Overall, the results of this study show that LNP-delivered TALEN-encoding mRNA has potentially curative use for HBV therapy. Augmenting the good efficacy of TALENs by using combinatorial therapy is an additional promising line of investigation.

## Data Availability

The original contributions presented in this study are included in the article and Supplementary Materials. Further inquiries can be directed to the corresponding author.

## Supplementary Materials

The following supporting information can be downloaded at: https://www.mdpi.com/article/10.3390/v17081090/s1, Figure S1: Confirmation of in vitro transcribed TALEN mRNA. Figure S2: Cell viability following TALEN mRNA transfection. Figure S3. Relative light units of reporter gene expression in transgenic mice. Table S1: Physicochemical properties of LNP formulations.

## Abbreviations

The following abbreviations are used in this manuscript:

HBV	Hepatitis B virus
cccDNA	Covalently closed circular DNA
ZFNs	Zinc finger nucleases
TALENs	Transcription activator-like effector nucleases
HBsAg	Hepatitis B surface antigen
HCC	Hepatocellular carcinoma
rcDNA	Relaxed circular DNA
C	*Precore*/*core*
Pol	*Polymerase*
S	*Surface*
ORFs	Open-reading frames
NAPs	Nucleic acid polymers
DSBs	Double-strand breaks
PAM	Protospacer adjacent motif
NHEJ	Non-homologous end joining
iLNPs	Ionizable lipid nanoparticles
IVT	In vitro transcription
HA	Hemagglutinin
NLS	Nuclear localization signal
UTR	Untranslated region
FLuc	Firefly luciferase
m1Ψ	N-methylpseudouridine
DMEM	Dulbecco’s Modified Eagle’s Medium
FBS	Fetal bovine serum
AAV2-D3	Adeno-associated virus serotype 2-subgenotype D3
MOI	Multiplicity of infection
PEG	Polyethylene glycol
N/P	Nitrogen-to-phosphorus ratio
FVB	Friend leukemia virus B
ALT	Alanine aminotransferase
AST	Aspartate aminotransferase
NHLS	National Health Laboratory Service
VPEs	Viral particle equivalents
pgRNA	Pregenomic RNA
Poly I:C	Polyinosinic: polycytidylic acid
TNF-α	Tumor necrosis factor-α
MCP-1	Monocyte Chemoattractant Protein-1
IL	Interleukin
SEM	Standard error of the mean
RLU	Relative light units
NTCP	Sodium taurocholate co-transporting polypeptide
