# Supplementary material for: Lipid Nanoparticle-Encapsulated TALEN-Encoding mRNA Inactivates Hepatitis B Virus Replication in Cultured Cells and Transgenic Mice
Source: Viruses. 2025 Aug 7;17(8):1090. doi: 10.3390/v17081090 (PMC12390527; doi:10.3390/v17081090)
Supplement: Supplementary file 1 [file viruses-17-01090-s001.zip › viruses-3742422-supplementary.pdf]

Supplementary Data:

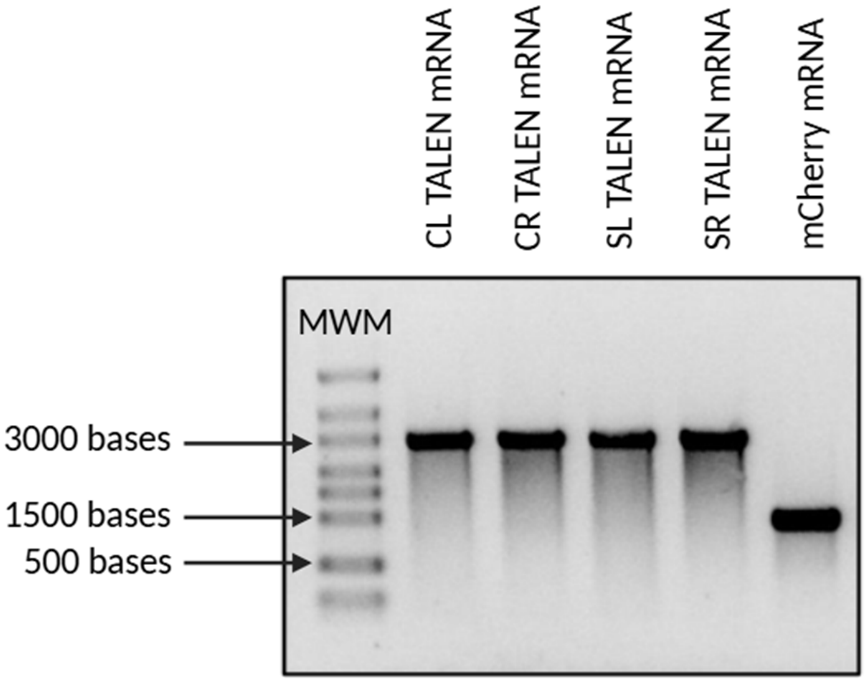

Figure S1: Confirmation of *in vitro* transcribed TALEN mRNA. *In vitro* transcribed transcripts were resolved on a 1% formaldehyde agarose gel to assess integrity and size. TALEN transcripts migrated at approximately 3 kb and mCherry migrated at approximately 0.8 kb. MWM: Molecular weight marker.

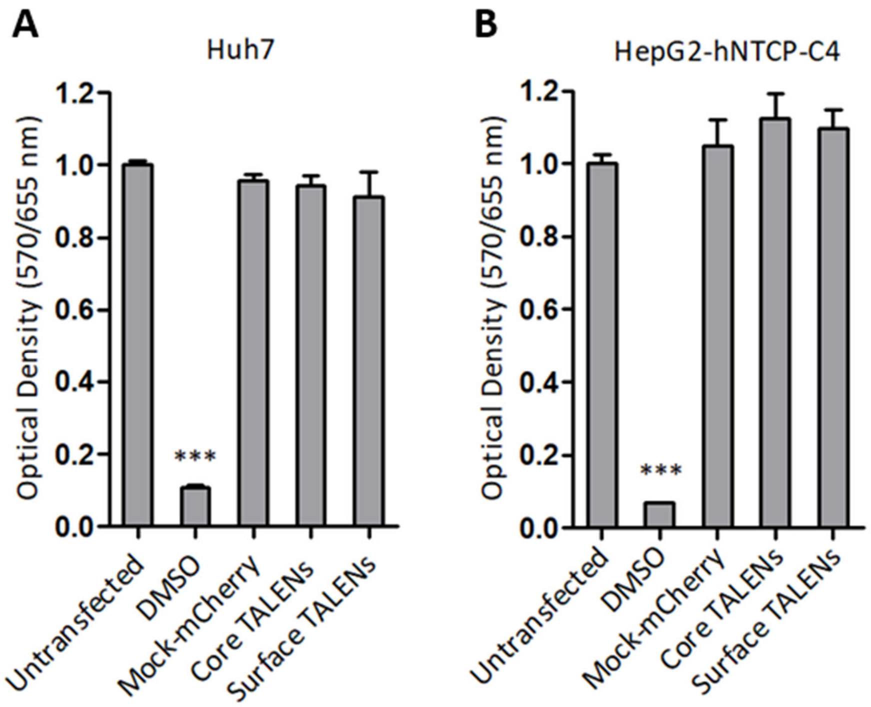

Figure S2: Cell Viability following TALEN mRNA transfection. MTT-based cell viability assay in Huh7 and HepG2-hNTCP-C4 cells transfected with TALEN mRNA or mock control mRNA. Cells treated with 50% DMSO served as a positive control. MTT reduction was measured 24 hours post-transfection. Data are shown as mean values ± SEM, with statistical significance compared to the positive control (***: $p<0.001$).

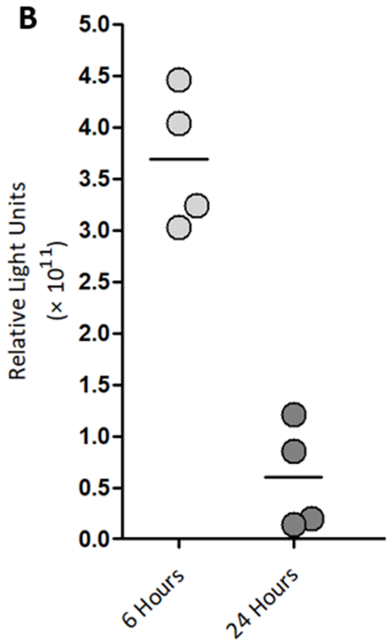

Figure S3. Relative light units of Reporter Gene Expression in Transgenic Mice. Relative luminescence units (RLU) for individual mice are shown at 6 and 24 hours post-injection.

Table S1: Physicochemical Properties of LNP Formulations

mRNA sequence	Size (nm)	Polydispersity index	Zeta potential (mV)	Encapsulation efficiency (%)
FLuc	88.3	0.1048	+8.103	93.99
mCherry	66.57	0.03811	-1.665	95.63
Core Left TALEN	72.77	0.08846	+7.904	96.65
Core Right TALEN	73.02	0.08989	+8.812	96.56
Surface Left TALEN	78.18	0.1108	+6.038	96.38
Surface Right TALEN	71.82	0.05677	+7.46	96.64
